# Adult intussusceptions caused by a lipoma in the jejunum: report of a case and review of the literature

**DOI:** 10.1186/1749-7922-7-28

**Published:** 2012-08-22

**Authors:** Ouadii Mouaqit, Hafid Hasnai, Leila Chbani, Bachir Benjelloun, Hicham El Bouhaddouti, Karim Ibn el Majdoub, Imane Toughrai, Said Ait Laalim, Abdelmalek Oussaden, Khalid Maazaz, Afaf Amarti, Khalid Ait Taleb

**Affiliations:** 1 Surgery Department, University Hospital Hassan II, Fez 30000, Morocco; 2 Department of pathology, University Hospital Hassan II, Fez, 30000, Morocco

**Keywords:** Intussusceptions, Jéjunal lipoma, Intestinal tumor, Surgery

## Abstract

Intussusceptions in adults is rare. Gastrointestinal lipomas are rare benign tumors and intussusceptions due to a gastrointestinal lipoma constitutes an infrequent clinical entity. Lipoma may develop as a benign tumor in all organs and rarely in large or small intestine. The present report describes a case of jejunojejunal intussusceptions in an adult with a history of colicky upper abdominal pain. Ileo-ileal invagination was diagnosed by computed tomography scan. Exploratory laparotomy revealed jejunojejunal intussusceptions secondary to a lipoma which was successfully treated with segmental intestinal resection. A review of the literature is also performed regarding this rare association revealing the diagnostic and therapeutic debates that exist.

**Abstract (french):**

L’invagination chez les adultes est rare. Les lipomes gastro-intestinaux sont de rares tumeurs bénignes et l’invagination intestinale due à un lipome gastro-intestinal constitue une entité clinique trés rare. Le lipome peut se développer comme une tumeur bénigne dans tous les organes et rarement dans l’intestin grêle ou le colon. Le présent rapport décrit un cas d’invagination jéjunojéjunale chez un adulte avec une histoire de douleurs abdominales. Iléo-iléale invagination a été diagnostiquée par tomodensitométrie. Une laparotomie exploratrice a révélé l’existence d’une invagination jéjunojéjunale secondaire à un lipome qui a été traitée avec succès par une résection intestinale segmentaire. Une revue de la littérature est également effectuée au sujet de cette association rare révélant les débats diagnostiques et thérapeutiques qui existent.

## Background

Intussusceptions was reported for the first time in 1674 by Barbette of Amsterdam [1]. The occurrence of intussusceptions in adults is rare, accounting for less than 5% of all cases of intussusceptions and almost 1%-5% of bowel obstruction [2]. In contrast to pediatric intussusceptions, which is idiopathic in 90% of cases, adult intussusceptions has an organic lesion in 70% to 90% of cases [3]. The majority of lipomas in the small bowel are solitary. Approximately 5% are multiple [4]. Symptomatic lipoma manifestations are hemorrhage or intestinal obstruction. Due to their intramural location, lipomas can also serve as the leading point for intussusceptions. We report a rare case of jejuno-jejunal intussusceptions in an adult secondary to an jejunal lipoma.

## Case presentation

**Figure 1:**
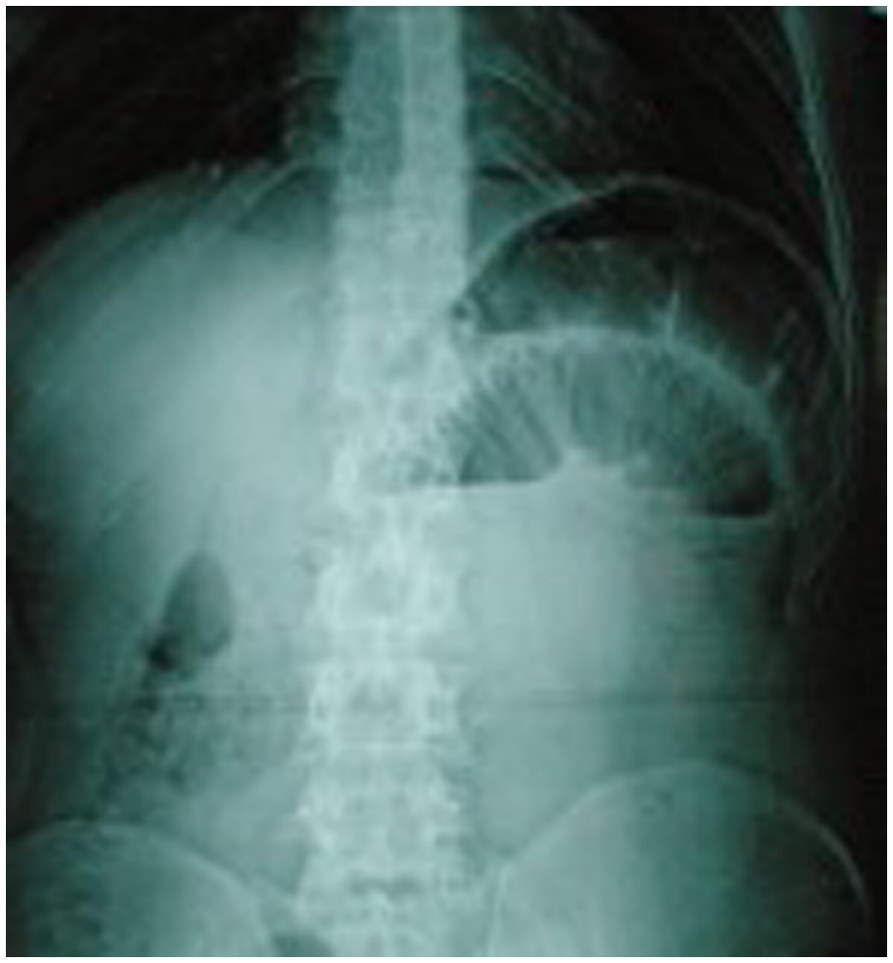
**Abdominal X-Ray.** In favor of bowel obstruction.

**Figure 2:**
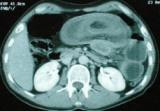
**Abdominal computed tomography.** Showing a fatty oval mass in the small intestine.

**Figure 3:**
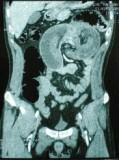
**Computed tomography scan of the abdomen without oral contrast.** A longitudinal cut view of the intussusception shows the “sausage” shape.

**Figure 4:**
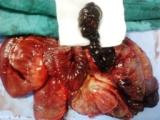
Intraoperative findings of the lipoma: A pedunculated lesion, measuring 60 mm, was the lead point of the intussusception.

**Figure 5:**
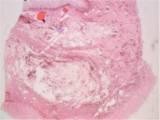
**Histological findings of the tumor.** A histopathologic examination of the tumor revealed fat cells proliferating in the submucosal layer.

A 35-year-old man was admitted to the emergency department in a tertiary referral hospital with 4 months history of intermittent upper abdominal pain accompanied with nausea. The patient had no past history of peptic ulcer disease, alteration in bowel habits, melena or weight loss. On examination, he was apyrexial and hemodynamically stable. His abdomen was distended and no palpable abdominal masses; bowel sounds were hyper audible. Initial A rectal examination revealed no masses or blood. Laboratory blood tests were normal. Abdominal radiography revealed prominent dilatation of the small bowel with air fluid levels (Figure 1). Abdominal CT showed a target sign- or sausage-shaped lesion typical of an intussusceptions that varied in appearance relative to the slice axis (Figure 2). The inner central area represented the invigilated intussuscepted, surrounded by its mesenteric fat and associated vasculature, and all surrounded by the thick-walled intussuscipiens. More head-side scans showed a low-density homogenous mass measuring 4 cm that was considered to be the leading point for the invagination (Figure 3). These findings led to a diagnosis of intussusceptions induced by a tumor most likely begin. The decision was made to undertake an urgent exploratory laparotomy. At laparotomy, 50 cm distal to the ligament of Treitz, a jejuno-jejunal intussusceptions was identified. We conducted a desinvagination Benin saw the character of the lesion on CT. The presence of irreversible ischemia in a small portion of the intussusceptum necessitated segmental resection and primary anastomosis (Figure 4). The postoperative period was uneventful and the patient was discharged on the sixth postoperative day. Gross examination of the respected specimen revealed a round tumor covered with mucosa measuring 6 cm. A microscopic examination revealed fat cells proliferating in the submucosal layer and confirmed the diagnosis of ileal lipoma (Figure 5). The histopathology report confirmed a 60-mm submucosal lipoma in the jejunum as a cause for a 30-cm jejuno-jejunal intussusceptions. There was no evidence of dysplasia or malignancy.

## Discussion

Intussusceptions in adulthood is unusual, with an incidence of approximately 2-3 cases per population of 1 000 000 per year [5]. The most common classification system divides intussusceptions into four categories: enteric, ileocolic, ileocaecal and colonic [1-4]. In adults, intussusceptions is more likely to present insidiously with vague abdominal symptoms and rarely presents with the classic triad of vomiting, abdominal pain and passage of blood per rectum, making diagnosis difficult [6]. Tumors of the small bowel account for only 1% to 2% of all gastrointestinal tumors, and benign tumors account for approximately 30% of all small-bowel tumors [7]. Lipomas are benign tumors of mesenchymal origin. They are the second most common benign tumors in the small intestine and account for 10% of all benign gastrointestinal tumors and 5% of all gastrointestinal tumors [1,2,5]. Gastrointestinal lipomas are most commonly located in the colon (65% to 75%), small bowel (20% to 25%) and occasionally in the foregut (< 5%) [8]. Fifty-one cases of adult intussusceptions induced by a lipoma, including our present case, have been reported in the English literature during the past decade (Table 1) [9]. Lipomas are largely asymptomatic. The majority of presenting features are either intestinal obstruction or hemorrhage [1,2,5-8]. Their usual location in the small intestine is ileum (50%) while jejunum is the least common. The peak age of incidence is in the 6th-7th decades of life and it appears that females are more prone to lipomas. Malignant degeneration has never been reported [5]. The clinical presentation is very non-specific which makes this a difficult condition to diagnose. According to the literature, only 32% to 50% of cases are diagnosed preoperatively, despite the evolution of imaging methods [9-11]. Abdominal pain, nausea, diarrhea and bleeding per rectum are the common symptoms. Rarely, this can present with acute intestinal obstruction. The classical triad of abdominal pain, sausage shaped palpable mass and passage of red current jelly stools seen in children is rarely seen in adults. Fewer than 20% of cases present acutely with complete bowel obstruction. A palpable abdominal mass is present in only 7% to 42% of cases [12,13]. Lipomas can be diagnosed through conventional endoscopy, capsule endoscopy, barium studies and, most importantly, CT scan [14]. Ultrasound is usually the first modality to be recruited. However, it is operator-dependent and the presence of distended bowel decreases the ability to demonstrate the site of the obstruction. Computed tomography is the imaging method of choice for diagnosing intussusceptions. A submucosal lipoma can be diagnosed if a smooth well-circumscribed mass of fat density (-50 to -100 Hounsfield Units) is revealed within the lumen of the bowel or intussuscipiens. The sensitivity of CT scan to correctly diagnose intussusceptions has been reported from 71.4%-87.5% while its specificity in adults has been reported to be 100% as verified by the subsequent surgery [14,15]. Capsule endoscopy and digital balloon endoscopy are newer means for diagnosing lipomas and are particularly helpful in cases involving small bowel lipomas [8]. Definitive surgical resection remains the recommended treatment for adult intussusceptions due to the large proportion of structural causes and the relatively high incidence of malignancy; however, the optimal surgical management remains controversial [1,2,6,7,9]. Some investigators have stated that small bowel intussusceptions should still be reduced only in patients in whom a definitive benign diagnosis has been made preoperatively, or in patients in whom resection may result in short gut syndrome [9]. The dangers of transperitoneal, vascular, and intraluminal seeding after exposing and handling friable and edematous malignant tissues has led many surgeons to advocate en bloc resection of the lesion. All surgeons agree, though, that reduction should not be attempted if there are signs of irreversible bowel ischemia, inflammation or when malignancy is being suspected [5,16,17]. The advantages of intraoperative reduction of the intussusceptions prior to resection, especially when the small bowel is affected, are that it may preserve a considerable length of bowel and thereby prevent development of short-bowel syndrome.

**Table 1:** The characteristics of the reported cases of adult intussusception induced by a lipoma

**Case**	**Age**	**Gender**	**Diagnostic modality**	**Tumor location**	**Size (cm)**	**Reference**
1	69	Male	US, CS	Descending colon	4	J Clin Ultrasound
2	42	Male	CS, BE, CT	Descending colon	4.5	Am Surg
3	39	Male	US, CT	Ileum	4	J Korean Med Sci
4	72	Male	EGD, US, CT	Stomach	10	Dig Surg
5	28	Male	CT	Jejunum	3	Ann R Coll Surg Engl
6	20	Female	CT	Ileum	18	Emerg Radiol
7	41	Male	CT	Ileum	ND	Australas Radiol
8	44	Female	CT, CS, ECS	Ileum	5	Abdom Imaging
9	51	Female	US, ECS, CT	Cecum	10	Rom J Gastroenterol
10	56	Male	US, CT	Ascending colon	6	J Laparoendosc Adv Surg Tech A
11	50	Male	ECS, CS, CT	Ascending colon	5	Pathol Int
12	72	Male	CT, EGD	Stomach	6	Can J Gastroenterol
13	55	Male	CT	Ileum	ND	Surg Today
14	63	Female	US, CT	Ileum	2.5	Surg Today
15	73	Female	ECS, MRI	Sigmoid colon	ND	Arch Surg
16	63	Male	CT	Ileum	3	JSLS
17	85	Male	US, CT	Jejunum	4	J Gastroenterol Hepatol
18	62	Male	CT, CS	Sigmoid colon	3.5	Dig Dis Sci
19	55	Female	CT	Transverse colon	12	Am Surg
20	31	Female	CT	Ascending colon	5	Can J Surg
21	47	Female	US, CT	Ileum	5	Ulus Travma Acil Cerrahi Derg
22	56	Female	US, CS, CT	Transverse colon	5	Ulus Travma Acil Cerrahi Derg
23	64	Male	CS, CT	Transverse colon	6	Clin Gastroenterol Hepatol
24	55	Male	CT, ECS	Jejunum	4	World J Gastroenterol
25	42	Male	US, CT	Ileum	3	Case Rep Gastroenterol
26	47	Female	CT	Ileum	3	J Laparoendosc Adv Surg Tech
27	47	Female	CT, CS, Enema	Ascending colon	5	Endoscopy
28	36	Male	CS, CT, ECS	Ileum	9	Cases J
29	36	Male	CT, ECS	Ileum	4	J Nippon Med Sch
30	82	Male	CS, CT	Sigmoid colon	8	Gastrointest Endosc
31	69	Male	CT, CS	Transverse colon	7	Dig Dis Sci
32	38	Female	CS, CT	Ileum	3.3	Clin Gastroenterol Hepatol
33	38	Female	US, CT, CS	Cecum	6	Emerg Radiol
34	45	Male	CT	Ileum	2.5	N Engl J Med
35	43	Female	CS, CT	Ascending colon	5	Rev Esp Enferm Dig
36	57	Female	CS, CT	Transverse colon	5.5	Rev Esp Enferm Dig
37	51	Male	US, CT, CS	Ileum	3	Gastroenterology
38	77	Male	CT	Cecum	3.5	JSLS
39	46	Male	CS, CT, ECS	Descending colon	6	Endoscopy
40	33	Male	CT, CS, BE	Ileum	4	Case Rep Gastroenterol
41	32	Female	CT	Ascending colon	5.8	Gastroenterology
42	49	Male	US, CT	Descending colon	5	Gastroenterology
43	53	Female	US, CS, ECS	Ascending colon	7	Medicina (Kaunas)
44	26	Female	CT	Ileum	ND	Am J Surg
45	51	Female	CT	Transverse colon	6.2	J Gastroenterol Hepatol
46	68	Male	CS	Jejunum	3.2	World J Gastroenterol
47	52	Female	CT	Ileum	3.2	J Med Case Reports
48	62	Female	US	Ileum	7	J Clin Ultrasound
49	65	Male	CT	Ileum	1.2	World J Gastrointest Surg
50	68	Female	US, CT, ECS	Ileum	1.5	Surg Today
51	35	Male	CT	jejunum	6	

## Conclusion

The lipoma is a rare benign tumor of the digestive tract. The diagnosis of intussusceptions in adults can be difficult because of atypical and episodic symptoms. A high level of clinical suspicion and an abdominal CT scan are most useful tools for making a timely diagnosis. Surgical resection remains the treatment of choice and produces an excellent prognosis.

## Consent

Written informed consent was obtained from the patient for publication of this case report and accompanying images

## Abbreviations

CT: Computed tomography; MRI: Magnetic resonance imaging; CS: Colonoscopy; ECS: Enema contrast study; EGD: Esophagogastroduodenoscopy; US: Ultrasonography; ND: Not described.

## Competing interests

The authors declare that they have no competing interests.

## Authors’ contributions

All of the authors were involved in the preparation of this manuscript. OM performed the operation and revised the manuscript. HH was an assistant surgeon and made substantial contributions to conception and design. LC described histological finding and was involved in drafting the manuscript. All authors read and approved the final manuscript.
